# Improved Perceptual Loss for Sketch Image Domain

**DOI:** 10.3390/jimaging11090323

**Published:** 2025-09-21

**Authors:** Chang Wook Seo

**Affiliations:** Anigma Technologies, Seoul 06241, Republic of Korea; lgtwins@anigma-ai.com

**Keywords:** computer vision, generative models, sketch image

## Abstract

Traditional perceptual loss functions, primarily designed for photographic images, often perform poorly in the sketch domain due to significant differences in visual representation. To address this domain gap, we propose an improved perceptual loss specifically designed for sketch images. Our method fine-tunes a pre-trained VGG-16 model on the ImageNet-Sketch dataset while strategically replacing max-pooling layers with spatial and channel attention mechanisms. We comprehensively evaluate our approach across three dimensions: generation quality, sketch retrieval performance, and feature space organization. Experimental results demonstrate consistent improvements across all evaluation metrics, with our method achieving the best generation performance, over 10% improvement in sketch retrieval accuracy, and 6-fold improvement in class separability compared to baseline methods. The ablation studies confirm that both fine-tuning and attention mechanisms are essential components that work together effectively. Our domain-specific perceptual loss effectively bridges the gap between photographic and sketch domains, providing enhanced performance for various sketch-related computer vision applications, including generation, retrieval, and recognition.

## 1. Introduction

Many generative methods for images have been proposed in computer graphics and computer vision. Traditional generative models, primarily designed to handle photographic images, often perform poorly when applied to other domains such as sketches. This discrepancy, known as the "domain gap," significantly reduces the effectiveness of these models in diverse applications. Our paper delves into this issue, particularly in the sketch image domain.

Our investigation focuses on enhancing perceptual loss [1] by considering the characteristics of sketch images. Perceptual loss is widely used in neural network training, particularly in image generation tasks. It focuses on minimizing the difference in high-level features and content between a generated image and a target image, as perceived by a pre-trained VGG-16 model [2], instead of focusing on pixel-level accuracy. By reconfiguring the architecture of the VGG-16 model and fine-tuning it using sketch images, we have developed a method that significantly improves the model’s performance across multiple sketch-related tasks.

In summary, this paper presents a new perceptual loss function to enhance both the quality of generated images and feature representation capabilities in the sketch image domain. This improved perceptual loss function effectively bridges the domain gap often encountered by existing models when dealing with sketch images. Through comprehensive evaluation across three dimensions—generation quality, sketch retrieval performance, and feature space organization—we demonstrate that our method not only improves sketch generation but also learns more semantically meaningful representations. We envision that the proposed method will be a meaningful addition to various sketch-related computer vision tasks, considering the rapidly increasing demand for applications involving sketch images such as webtoons, digital art, visual effects, and sketch-based image retrieval tasks.

## 2. Related Work

### 2.1. Generative Methods in the Sketch Domain

Including deep learning-based methods, there are numerous image generation methods, such as image translation [3], neural style transfer [1,4,5], StyleGAN [6], and so on. Many of these methods primarily deal with photorealistic images and are unable to handle sketch images, which have significantly different visual representations.

To address this domain gap, methods that are specifically designed to handle sketch images have been proposed. Early approaches focused on traditional sketch processing and domain adaptation. Simo et al. [7] introduced an image translation method that can convert rough sketches into clean sketches. Seo et al. [8] presented a method for extracting sketches from color images. For adaptation to the sketch image domain, Xu et al. [9] fine-tuned a VGG-16 model [2] on sketch images by improving a perceptual loss function for model optimization.

Building upon these foundational studies, diffusion-based methods have recently advanced the field of sketch generation to a new level. For example, CoProSketch [10] introduces a controllable and progressive diffusion framework that leverages unsigned distance fields along with user-editable feedback loops. Meanwhile, DiffSketcher [11] adapts pre-trained text-to-image diffusion models to produce vectorized freehand sketches through curve optimization and score distillation sampling. More recently, StrokeFusion [12] proposes a two-stage framework combining stroke-UDF encoding with latent sequence diffusion, enabling high-fidelity vector sketch generation with support for stroke interpolation editing. Together, these approaches highlight a growing shift toward diffusion techniques as a means of achieving higher fidelity, greater controllability, and more expressive abstract sketch representations.

Moreover, related studies [13,14,15,16,17,18,19] utilize large model priors to generate sketch images through their novel adaptations. These approaches leverage pre-trained models to better align with the characteristics of the sketch domain. These methods commonly tailor the generative process or feature representation to enhance fidelity and controllability in sketch synthesis.

### 2.2. Attention in Image Processing

Attention mechanisms are well known for their effectiveness in enhancing deep learning-based methods where they are applied to find and extract important features from the input. Various types of attention mechanisms exist across different domains, including natural language processing and computer vision. In this paper, we employ spatial and channel attention mechanisms, which are specifically designed to extract important features from images in terms of both content and style.

Spatial attention focuses on extracting features in the spatial embedding, which is closely related to the content of the image, while channel attention concentrates on features in the channel embedding, which is closely related to the style of the image. Building on these principles, previous studies [4,5,8] have utilized both types of attention mechanisms to extract individual style and content features effectively, resulting in high-quality fashion image generation. Furthermore, Seo et al. [8] employed these features to extract information from an input color image and a style reference image, allowing them to imitate the style of the reference image in the output sketch image while preserving the content of the input color image.

## 3. Method

Our method improves the perceptual loss for the image translation tasks of sketch images in two ways.

- Using the ImageNet-Sketch [20] dataset to fine-tune the pre-trained VGG-16 model.
- For the model tuning process, replacing the 5th and 10th layers, which were originally max-pooling layers, with spatial and channel attention layers in the VGG-16 model.

As mentioned in Section 2.1, tuning a pre-trained ImageNet model in a designated image domain has been shown to yield improved performance [9]. Inspired by this previous work, we fine-tuned the pre-trained VGG-16 model using a large-scale sketch dataset, ImageNet-Sketch [20], to create our model.

**Figure 1:**
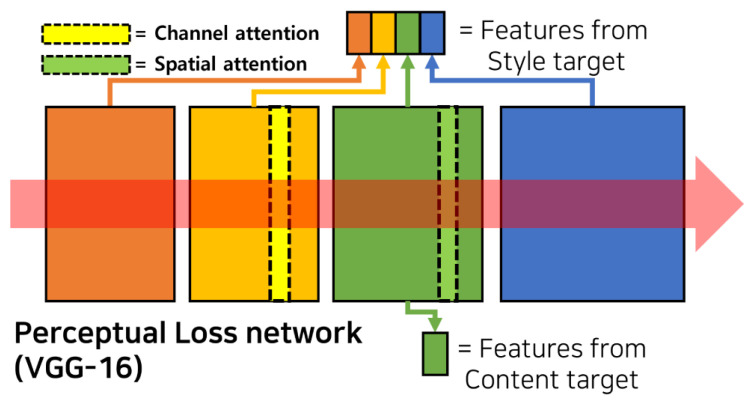
Max-poolinglocated at the 5th and 10th layers are replaced by channel and spatial attentions in the VGG-16 model.

For the fine-tuning, we made additional changes to enhance the performance and optimize the training for sketch images. Inspired by Seo et al. [8] and Ashtari et al. [21], who applied image processing methods to sketch images, we added spatial and channel attention to the pre-trained VGG-16 model. The attention layers are placed in the 5th and 10th layers of the VGG-16 model, where the max-pooling layers were originally located. See Figure 1 for illustration. These attention structures are akin to those employed in the Convolutional Block Attention Module (CBAM) [22]. The details of these attentions are described below.

Consider an input feature map denoted as $i\in R^{C\times H\times W}$. Here, *C* indicates the number of channels, *H* is the height, and *W* is the width of the image. We treat spatial and channel attentions separately for computation. Specifically, the spatial attention is denoted by $SP_{a}\in R^{1\times H\times W}$ and the channel attention is denoted by $CH_{a}\in R^{C\times 1\times 1}$.

(1) $$\begin{array}{c} \begin{array}{cc} \; & SP_{a}\left(E_{c}\left(C_{i}\right)\right)\\ \; & =\sigma (f^{7\times 7}\left(\left[{\left(i\right)}_{avg}^{sp};{\left(i\right)}_{max}^{sp}\right]\right)),\; \end{array} \end{array}$$

(2) $$\begin{array}{c} \begin{array}{cc} \; & CH_{a}\left(\left(i\right)\right)\\ \; & =\sigma (W_{1}\left(W_{0}{\left(i\right)}_{avg}^{ch})\right)+W_{1}\left(W_{0}{\left(i\right)}_{max}^{ch})\right)), \end{array} \end{array}$$

In Equation (1), the features from *i* undergo two distinct pooling functions before they are convolved with a 7×7 kernel filter. In Equation (2), the features from input *i* undergo two distinct pooling functions prior to passing through Multi-Layer Perceptron (MLP) layers. The weights of these MLP layers are denoted as $W_{0}\in R^{C/r\times 1}$ and $W_{1}\in R^{C\times C/r}$, where a reduction ratio of r=16 is applied. For both types of attention mechanisms, sigmoid functions σ are utilized. The sizes of the features after the pooling layers are denoted as $AvgPool^{ch},MaxPool^{ch}\in R^{C\times 1\times 1}$ for channel attentions, and $AvgPool^{sp},MaxPool^{sp}\in R^{1\times H\times W}$ for spatial attentions.

The model was trained for 25 epochs with a batch size of 32 and an Adam optimizer without weight decay. Data augmentation included random resized cropping to 224×224, random horizontal flipping, and normalization with ImageNet statistics. The best model was selected based on validation accuracy and saved for subsequent experiments. See Algorithm 1 for the training details.
**Algorithm 1**
Overview of the training pipeline
  1:**Input:** ImageNet-Sketch dataset D  2:**Preprocessing:** Perform RandomResizedCrop(224 × 224), RandomHorizontalFlip, and normalize using ImageNet mean and variance  3:**Data Partition:** Split D into 90% training set ($D_{train}$) and 10% validation set ($D_{val}$)  4:**Model Setup:** Initialize pretrained VGG-16, substituting the 5th and 10th max-pooling layers with attention modules  5:**Optimization:** Train with Adam optimizer (η=0.005), batch size of 32, for 25 epochs  6:Initialize variable best_val_acc ←0  7:**for** epoch=1 to 25 **do**  8:      Run forward pass on $D_{train}$, compute classification loss  9:      Update model parameters via backpropagation10:    Evaluate on $D_{val}$, obtain validation accuracy11:    **if** current validation accuracy > best_val_acc **then**12:          Store current model parameters as the best checkpoint13:          Update best_val_acc14:    **end if**15:**end for**16:**Output:** Best-performing model weights selected by validation accuracy

## 4. Experiments

To validate the effectiveness of our improved perceptual loss, we conduct comprehensive evaluations across multiple dimensions: (1) sketch generation quality through ablation studies, (2) feature representation quality through sketch retrieval experiments, and (3) feature space analysis through intra/inter-class distance measurements.

### 4.1. Generation Quality Analysis

To evaluate the effectiveness and generality of our improved perceptual loss function, we conduct experiments using two fundamentally different generative paradigms: (1) a classical perceptual-style transfer model [1], and (2) a modern latent diffusion [23] model based on Stable Diffusion. This dual framework evaluation allows us to test both the fidelity and the adaptability of our method across diverse generation pipelines, ranging from image-to-image to prompt-to-image synthesis.

#### 4.1.1. Experimental Settings

**Style transfer model (image-to-image)**

For the style transfer baseline, we adopted the model proposed by Johnson et al. [1], which is a widely recognized framework for neural style transfer based on perceptual loss. This model directly incorporates perceptual loss for optimizing stylized image generation, and has been established as a benchmark in the literature [24].

We trained this model using the COCO dataset [25] (118,288 images) as the content image source. For the style images, we used two sketch datasets: CUFS [26] and FS2K [27]. While CUFS is a well-known paired dataset for sketch-to-photo synthesis, FS2K is a more recent and diverse dataset containing three distinct sketch styles. These multiple sketch styles in FS2K, together with the stylistic differences of CUFS, provide a natural setting for evaluating artistic style variation, which we highlight in our experiments. We trained separate models for each dataset, and for FS2K, we further trained individual models for each sketch style. Training was performed for a single epoch with a batch size of 4, and all other settings followed the original implementation [1].

**Latentdiffusion model (Prompt-to-Image)**

To evaluate our method in a more recent and semantically driven setting, we fine-tuned the Stable Diffusion v1.5 [23] model on the ImageNet-Sketch [20] dataset using LoRA (Low-Rank Adaptation) [28]. Stable Diffusion was chosen as our baseline not only because it is one of the most widely adopted open-source text-to-image frameworks [23], but also due to its demonstrated robustness and extensibility in numerous follow-up works that build upon it, such as ControlNet [29], T2i-adapter [30], and LoRA-based fine-tuning methods [28], which all report their improvements by enhancing Stable Diffusion as the base model. Furthermore, focusing on Stable Diffusion alone allows us to conduct a consistent and fair evaluation, since comparing across heterogeneous diffusion architectures could introduce confounding factors. Given its central role as the foundation of many state-of-the-art extensions, validating our method on Stable Diffusion provides a strong and generalizable baseline.

In this setting, each sketch image is paired with a class-specific text prompt such as “a sketch of a Pembroke”, derived from the class name. This enables a prompt-to-image generation setup aligned with modern text-to-image workflows.

Inspired by Berrada et al. [31], we implemented a simplified perceptual loss approach by decoding denoised latents into RGB image space and comparing VGG feature activations via L1 loss, rather than using the more sophisticated internal decoder features proposed in their work. Training was conducted for 100 epochs using a batch size of 1 and a resolution of 256×256. Mixed precision (fp16) was employed for memory efficiency, and other hyperparameters such as learning rate, scheduler, and noise schedule followed standard Stable Diffusion fine-tuning practices.

#### 4.1.2. Evaluation

To evaluate the effectiveness of our improved perceptual loss, we conducted an ablation study by individually removing the attention modules and the tuning process. The choice of evaluation metrics was aligned with the input–output characteristics of each generative model.

For the Johnson et al. [1] model, which performs image-to-image translation, we adopted SSIM (Structural Similarity Index) and FID (Fréchet Inception Distance) as evaluation metrics. A higher SSIM and a lower FID indicate better reconstruction quality and greater similarity to the ground truth image.

In contrast, for the Stable Diffusion model, which generates images from text prompts (prompt-to-image), we employed CLIP similarity [32] and FID. CLIP score reflects the semantic alignment between the input prompt and the generated image, while FID measures overall image quality and distributional similarity to reference data.

For quantitative comparison, Table 1 presents the results for the style transfer model, while Table 2 presents the results of the latent diffusion model. Our new perceptual loss achieved consistently high scores across multiple methods and dataset styles, outperforming the average scores of other ablated alternatives.

**Figure 2:**
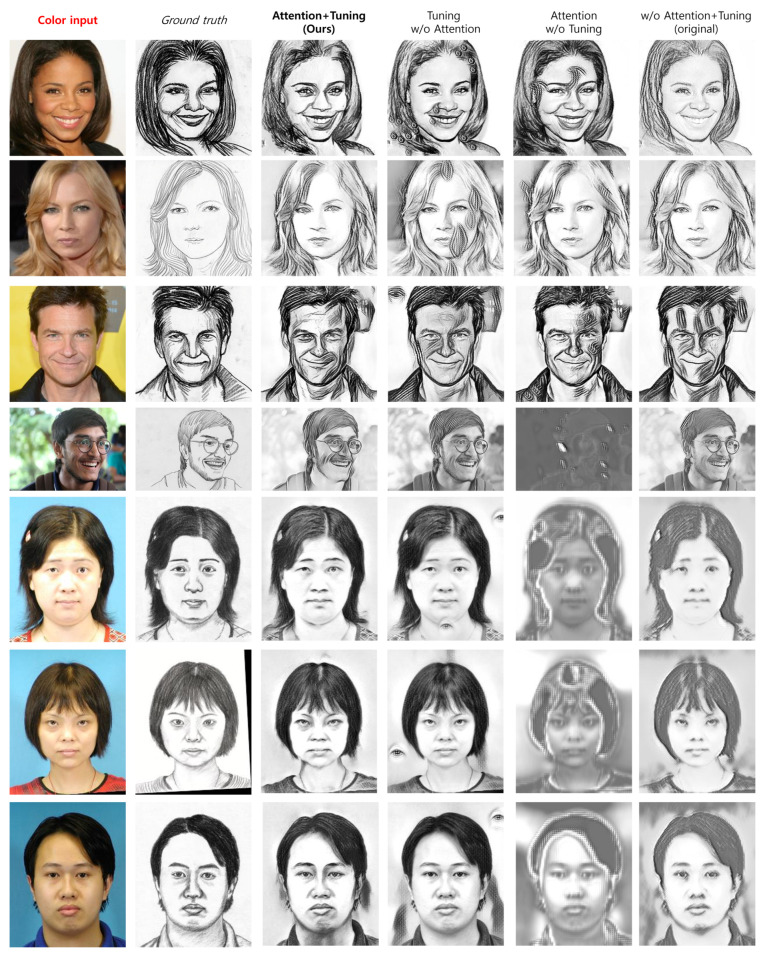
Examples of ablation study results obtained by removing the attention mechanism and the tuning process in style transfer. The images are from the CUFS [26] and FS2K [27] datasets.

**Figure 3:**
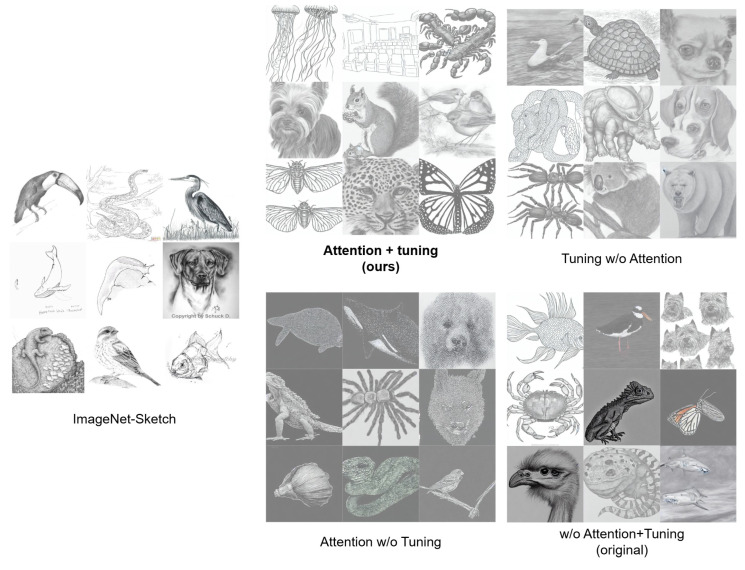
Examples of the ablation study results obtained by removing the attention and tuning process in the latent diffusion model. The model was trained on the ImageNet-Sketch [20] dataset.

For qualitative comparison, Figure 2 and Figure 3 show examples of generated images across different ablation settings. In the style transfer setting (Figure 2), our method achieved high-quality results without artifacts, with a drawing style that faithfully matches the ground truth sketches. When the model was fine-tuned without attention layers, it produced unnecessary details, such as eye textures, in certain areas. On the other hand, when the model with attention layers was not fine-tuned, the produced output quality was unpredictable, suggesting training failure. The use of the original perceptual loss, without any additional improvements, often failed to transfer the input image into a proper sketch and merely converted pixel colors to grayscale.

In the latent diffusion model setting (Figure 3), our method again produced outputs most similar to the original ImageNet-Sketch domain, successfully rendering clean sketch images on white backgrounds. Fine-tuning without attention layers retained some stylistic similarity but occasionally resulted in content deformation. When using attention layers without fine-tuning, the model entirely failed to optimize toward the sketch domain, leading to low-quality and inconsistent outputs. Finally, when neither attention layers nor fine-tuning were applied, the model could generate plausible images, but some results exhibited photographic color artifacts instead of sketch-like rendering.

Importantly, unlike the facial sketch dataset used in the style transfer setting, the ImageNet-Sketch dataset covers 1000 diverse object categories. Our improved perceptual loss consistently preserved sketch fidelity and semantic alignment not only for human faces but also across a wide range of non-facial classes. This demonstrates that the proposed loss function generalizes effectively to heterogeneous domains, reinforcing its robustness for large-scale sketch generation tasks.

### 4.2. Sketch Retrieval Experiment

Beyond generation quality, we evaluate whether our improved perceptual loss learns better feature representations for sketch understanding tasks. To this end, we conducted a sketch-based image retrieval experiment using the learned perceptual features.

#### 4.2.1. Experiment Setting

Given a query sketch, the goal is to retrieve the most similar sketch from a gallery set using cosine similarity over perceptual feature embeddings. We compare two different VGG-16-based models: (1) a baseline model using pretrained ImageNet features, and (2) a fine-tuned model trained on sketch data with both classification and triplet loss objectives.

The fine-tuned model employs a VGG-16 backbone with modified classifier layers that output 512-dimensional normalized features. During training, we freeze the feature extraction layers and only train the classifier components using a combination of cross-entropy loss for classification and triplet loss for metric learning with a margin of 0.2. The total loss is computed as $L_{total}=L_{CE}+0.01\cdot L_{triplet}$, where the triplet loss weight is reduced to balance the two objectives.

We used the ImageNet-Sketch dataset and split it into training (90%) and testing (10%) sets. The gallery consists of training images, while test images serve as queries. Training is performed for 20 epochs with the Adam optimizer (learning rate: 0.0001) and a learning rate scheduler that reduces the rate by 0.1 every 7 epochs. To ensure robustness, all experiments are repeated five times with different random seeds, and we report the average performance across these runs.

#### 4.2.2. Evaluation

Table 3 summarizes the results of the sketch retrieval experiment. Our perceptual loss achieves significantly higher retrieval performance, with improvements of more than 10% in both Top-1 Accuracy. This demonstrates that features extracted using our loss are more semantically aligned in the sketch domain, confirming that our improvements benefit not only generation quality but also feature representation capability.

### 4.3. Intra-Class vs. Inter-Class Distance Experiment

Feature quality evaluation through intra-and inter-class distance analysis is a well-established method in deep learning, particularly effective for assessing fine-tuned networks [33]. The separation ratio is defined as the ratio of inter-class to intra-class distance, which serves as a unified metric for measuring the discriminative capability of learned representations [34]. In the sketch domain, where perceptual similarity is nuanced, this approach enables direct comparison of the novelty of our pre-trained model compared to previous evaluations.

#### 4.3.1. Experiment Setting

**Figure 4:**
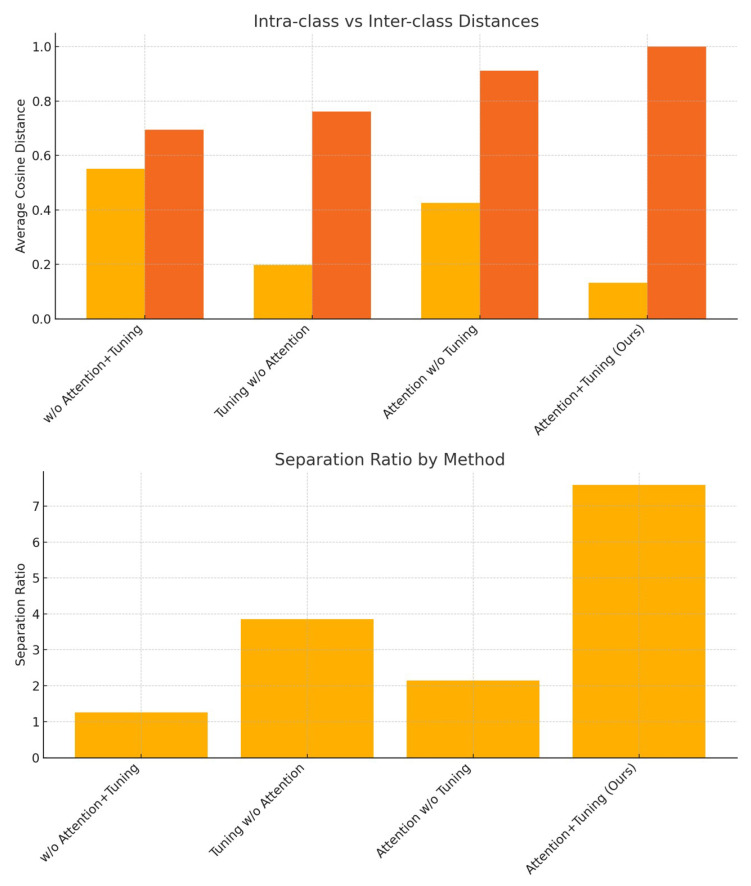
Graph which visualizes the separation ratio gap between ablation studies.

A desirable perceptual feature space should exhibit low intra-classdistances (tight clustering of similar sketches) and high inter-classdistances (clear separation between different identities), resulting in a high separation ratio. To evaluate how each loss function organizes the sketch feature space, we conducted an ablation study. Cosine distances were computed pairwise between all sketches within the same identity (intra-class) and across different identities (inter-class). For this experiment, we utilized the ImageNet-Sketch dataset without separating the training and test datasets. The average distances and the resulting separation ratios are reported in Table 4 and illustrated in Figure 4.

#### 4.3.2. Evaluation

Our results reveal several key insights. First, fine-tuning without attention already leads to substantial improvements: intra-class distances are reduced by 64.1% (from 0.551 to 0.198), and inter-class distances increase modestly by 9.6%, resulting in a 3.05× gain in separation ratio. Second, applying attention without tuning yields more limited improvements, primarily increasing inter-class distances (0.912 vs. 0.695) but less effectively reducing intra-class spread. The resulting separation ratio of 2.14 reflects this modest enhancement.

Notably, the complete method (**Attention+Tuning**) combines both benefits. It achieves the smallest intra-class distance (0.132) and the largest inter-class distance (1.000), producing a separation ratio of **7.59**, representing a **6.01× improvement** over the baseline. Compared to tuning alone, the attention mechanism further improves intra-class compactness and inter-class discrimination, validating its complementary role.

These findings indicate that fine-tuning is the primary implementation of discriminative feature learning, while attention mechanisms further refine the embedding space. The gains in separation ratio confirm that our perceptual loss formulation leads to a semantically meaningful organization of sketches. Sketches of the same identity are tightly clustered, while those of different identities are well separated. This translates to enhanced sketch retrieval and recognition, highlighting the general utility of our loss beyond generation-focused tasks.

## 5. Discussion

Our comprehensive evaluation across three perspectives: generation quality, retrieval performance, and feature space organization consistently demonstrates the novelty of our improved perceptual loss. The ablation studies reveal that both attention mechanisms and fine-tuning are essential components that work synergistically. The generation quality analysis in both style transfer and latent diffusion methods shows that our method achieves the best average performance. The sketch retrieval experiment provides compelling evidence that our method learns more semantically meaningful features, with over 10% improvement in retrieval accuracy. The distance analysis offers quantitative evidence of improved feature space organization.

## 6. Limitations and Future Work

While our work demonstrates the effectiveness of perceptual loss in both traditional neural style transfer and modern diffusion-based sketch generation, further exploration across diverse sketch domains and artistic styles would strengthen our findings. It is also important to note that our method is specifically designed for sketch images and does not claim novelty for photo image generation, which we explicitly acknowledge as a limitation. Contemporary sketch generation methods using transformer-based architectures [35,36,37] and specialized diffusion approaches [38,39,40] represent promising directions for integration. As future work, we plan to extend our evaluation to broader sketch domains and investigate adaptive weighting strategies between different loss components. Our perceptual loss framework, being architecture-agnostic, can potentially enhance various generative methods that incorporate perceptual objectives.

## 7. Conclusions

In this paper, we introduce a novel perceptual loss for improving both image quality and feature representation learning in the sketch image domain. Our method fine-tunes a VGG-16 model on the ImageNet-Sketch dataset while integrating spatial and channel attention layers to replace max-pooling operations. Our work demonstrates that sketch-domain-specific perceptual loss design can bridge the gap between the photographic and sketch domains.

## Figures and Tables

**Table 1:** Quantitative resultsof ablation studies in the style transfer model method [1]. Performed by removing the attention and tuning processes individually. The best SSIM scores are underlined while the best FID scores are **bolded**.

(SSIM/FID)	Attention+Tuning (Ours)	Tuning w/o Attention	Attention w/o Tuning	w/o Attention + Tuning (Original)
CUFS	0.5308/**92.06**	0.5049/119.84	0.4886/233.87	0.5173/105.76
FS2K_style1	0.4132/**65.66**	0.3703/100.15	0.4176/100.15	0.4216/70.16
FS2K_style2	0.2824/73.25	0.2396/77.60	0.2998/73.57	0.2770/**58.70**
FS2K_style3	0.5656/**126.66**	0.5454/168.16	0.4979/398.16	0.5618/150.00
**Average**	0.4480/**89.40**	0.4150/116.16	0.4259/201.43	0.4444/96.15

**Table 2:** Quantitative results of ablation studies in latent diffusion model method [23]. Performed by removing the attention and tuning processes individually. The best CLIP scores are underlined while the best FID scores are **bolded**.

	Attention+Tuning (Ours)	Tuning w/o Attention	Attention w/o Tuning	w/o Attention + Tuning (Original)
CLIP/**FID**	27.19/**65.56**	26.09/85.03	22.48/122.87	26.15/80.04

**Table 3:** Sketch retrieval accuracy (mean ± standard deviation) over five independent runs using different perceptual losses. Paired *t*-tests were conducted against the strongest baseline (Tuning w/o Attention); statistically significant improvements are marked with * (*p* < 0.05).

Method	Top-1 (%)	Top-3 (%)	Top-5 (%)
Attention+Tuning (Ours)	**74.90 ± 0.31** *	**83.51 ± 0.28** *	**87.75 ± 0.24** *
Tuning w/o Attention	73.74 ± 0.29	82.71 ± 0.34	85.36 ± 0.27
Attention w/o Tuning	59.01 ± 0.52	65.44 ± 0.48	70.10 ± 0.43
w/o Attention + Tuning	73.05 ± 0.33	81.13 ± 0.36	83.87 ± 0.31

**Table 4:** Average cosine distances of perceptual features between sketches (mean ± standard deviation) over five independent runs. Paired *t*-tests were conducted against the strongest baseline (Tuning w/o Attention); statistically significant improvements are marked with * (*p* < 0.05).

Method	Intra-Class	Inter-Class	Separation Ratio
w/o Attention+Tuning	0.551 ± 0.015	0.695 ± 0.013	1.26
Tuning w/o Attention	0.198 ± 0.014	0.762 ± 0.018	3.85
Attention w/o Tuning	0.426 ± 0.017	0.912 ± 0.020	2.14
Attention+Tuning (Ours)	0.132 ± 0.012 *	1.000 ± 0.041 *	7.59

## Data Availability

This study used only publicly available datasets. All data used are openly accessible from public sources as cited in the manuscript.
